# Mitophagy in gynecological malignancies: roles, advances, and therapeutic potential

**DOI:** 10.1038/s41420-024-02259-x

**Published:** 2024-12-05

**Authors:** Jiao Wang, Dandan Wang

**Affiliations:** Department of Obstetrics and Gynecology, Shengjing Hospital of China Medical University, Shenyang, 110004 China

**Keywords:** Gynaecological cancer, Gynaecological cancer

## Abstract

Mitophagy is a process in which impaired or dysfunctional mitochondria are selectively eliminated through the autophagy mechanism to maintain mitochondrial quality control and cellular homeostasis. Based on specific target signals, several mitophagy processes have been identified. Defects in mitophagy are associated with various pathological conditions, including neurodegenerative disorders, cardiovascular diseases, metabolic diseases, and cancer. Mitophagy has been shown to play a critical role in the pathogenesis of gynecological malignancies and the development of drug resistance. In this review, we have summarized and discussed the role and recent advances in understanding the therapeutic potential of mitophagy in the development of gynecological malignancies. Therefore, the valuable insights provided in this review may serve as a basis for further studies that contribute to the development of novel treatment strategies and improved patient outcomes.

## Facts

- The latest advances of mitophagy in the pathogenesis and development of gynecological malignancies.
- Understanding the regulatory mechanisms of mitophagy is crucial for unraveling the molecular basis of gynecological malignancies.
- The dysregulation of mitophagy has a potential link to the therapeutic resistance in gynecological cancers.

## Open questions

- What are the precise mechanisms of mitophagy in tumorigenesis and drug resistance in gynecological malignancies?
- Can the mitochondrial proteins or mitophagy-related proteins be used as biomarkers to predict the prognosis of gynecological malignancies?
- How to develop new effective drugs that target mitochondrial proteins or mitophagy-related proteins for the treatment of gynecological malignancies?

## Introduction

The most common gynecological malignancies are ovarian cancer (OC), cervical cancer (CC), and endometrial cancer (EC), which pose serious risks to female health. The predominant therapeutic approaches for these cancers include surgery, radiotherapy, chemotherapy, and targeted therapy. In recent years, there have been remarkable advancements in the screening, diagnosis, and treatment of gynecological malignancies [1–10]. Despite these improvements, the prognosis of patients remains poor, and the availability of curative options remains limited, particularly for patients with OC [2]. Therefore, it is essential to explore potential biomarkers and therapeutic targets to improve the prognosis of individuals affected by gynecological malignancies.

Mitochondria are double-membrane organelles known as the “powerhouses” of the cell and play critical roles in energy production [11, 12], calcium buffering [13, 14], oxidative stress [15–17], and various forms of regulated cell death, such as ferroptosis [18, 19] and apoptosis [18, 20]. The dynamic balance of the mitochondrial network is maintained by the constant fusion and fission of mitochondria, while the fusion process neutralizes damaged mitochondrial components and the fission process isolates damaged mitochondria for degradation [21–23].

Mitophagy, the selective recognition and degradation of dysfunctional or damaged mitochondria by autophagic machinery, is an indispensable mechanism in the quality control of mitochondria and cellular homeostasis [24, 25]. The process involves the selective sequestration of excess or impaired mitochondria in autophagosomes, which subsequently fuse with lysosomes, ultimately leading to mitochondrial degradation. Impaired mitophagy function is involved in the progression and pathogenesis of multiple diseases, including neurodegenerative conditions [26, 27], metabolic diseases [28], cardiovascular diseases [29, 30], autoimmune disorders [31, 32], and cancers [33–35]. Consequently, mitophagy exhibits considerable potential in the prevention and treatment of tumors.

Understanding the regulatory mechanisms underlying mitophagy is crucial for unraveling the molecular basis of various cancers and developing novel drugs that target mitochondrial proteins or mitophagy-related proteins. In this review, we present a thorough overview of our current understanding of the molecular pathways of mitophagy underlying the onset and progression of gynecological cancers, emphasizing the role of mitophagy as a precise therapeutic target in this setting.

## Overview of the major mitophagy pathways

The regulatory mechanisms of mitophagy have been mainly divided into ubiquitin-dependent and ubiquitin-independent or receptor-mediated mitophagy. In the following section, we focus on the various regulatory pathways of mitophagy.

### Ubiquitin-dependent mitophagy

Traditionally, ubiquitin-dependent mitophagy has primarily been associated with the PTEN-induced kinase 1 (PINK1)/Parkin mitophagy pathway, which has attracted considerable attention. This pathway typically involves the involvement of PINK1/PARK6, a serine/threonine kinase, and Parkin (PARK2), an E3-ubiquitin ligase [24]. However, emerging evidence has revealed that the ubiquitylation of mitochondrial proteins is not entirely dependent on Parkin. Several other E3-ubiquitin ligases have been identified that exhibit synergistic or substitutive effects with Parkin [36–41].

#### Parkin-dependent mitophagy

**Fig. 1:**
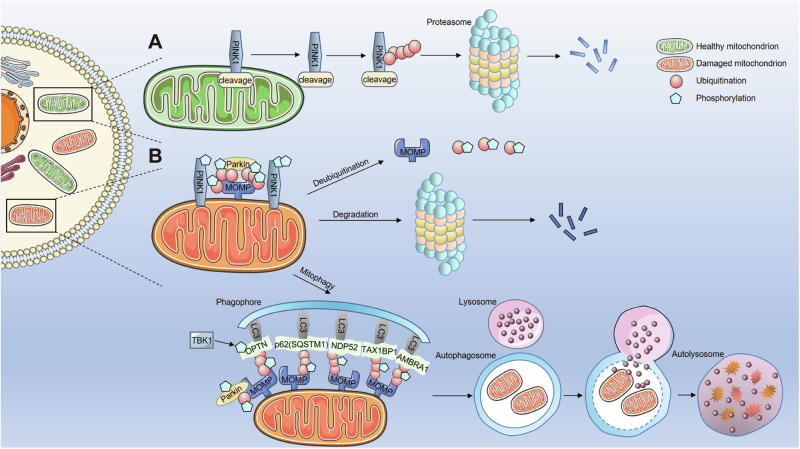
Fate of PINK1 in healthy mitochondria and the three fates of Parkin-ubiquitinated mitochondrial proteins in damaged mitochondria. **A** Under normal basal conditions, PINK1 is imported into the polarized mitochondria by utilizing the mitochondrial translocases located in the MOM and MIM. Subsequently, PINK1 undergoes two sequential cleavage events within the MIM, followed by proteasome-mediated degradation. **B** When mitochondria are damaged, full-length PINK1 is arrested and stabilized on the MOM, where it phosphorylates Ub and/or Parkin. Activated Parkin further drives Ub chain formation on the MOM proteins, which are then phosphorylated by PINK1. There are three fates of Parkin-ubiquitinated mitochondrial proteins in damaged mitochondria. First, the ubiquitinated proteins can be deubiquitinated by DUBs; second, the ubiquitinated proteins can be degraded by the ubiquitin-proteasome system; third, the recruitment of autophagy adaptors such as OPTN, NDP52, p62, TAX1BP1, and AMBRA1 by Parkin-conjugated polyubiquitin chains serve as an initiating mechanism for the delivery of mitochondria to autophagosomes; then autophagosomes fuse with lysosomes for their further degradation, which is the classic PINK1/Parkin-mediated mitophagy pathway.

The PINK1/Parkin-dependent mitophagy pathway, which is ubiquitin-dependent, was originally described by Narenda et al. [42]. In a healthy state, PINK1 is transported into the polarized mitochondria and is subsequently cleaved twice in the mitochondrial inner membrane (MIM) [43–46]. The cleaved PINK1 is then retro-translocated to the cytosol for proteasomal degradation [43, 45, 47] (Fig. 1A). Full-length PINK1 is stabilized and arrested on the mitochondrial outer membrane (MOM) under the condition of mitochondrial damage [45–47]. As PINK1 stabilizes on the MOM and autophosphorylates, it influences Parkin by phosphorylating pre-existing ubiquitin molecules already conjugated to MOM proteins at Ser65 [48], by phosphorylating pSer65-Ub-bound Parkin [44], or by directly phosphorylating the Parkin in the absence of its initial encounter with pSer65-Ub [44, 49, 50]. Owing to its high affinity for phosphorylated ubiquitin, Parkin is easily transported from the cytoplasm to the mitochondria. Simultaneously, Parkin’s activated E3-ubiquitin ligase activity promotes the formation of ubiquitin chains on MOM proteins, such as mitofusin 1 (MFN1), mitofusin 2 (MFN2), ras homolog family member T (RHOT/MIRO), voltage-dependent anion channel (VDAC), and BAK, thus forming a positive feedback loop that amplifies the original signal, leading to extensive Parkin recruitment and ubiquitination [44, 51, 52].

Parkin-ubiquitinated mitochondrial proteins undergo three distinct fates (Fig. 1B). First, deubiquitinases remove the ubiquitin moieties from the proteins, restoring their original state and preventing mitophagy [53–55]. Second, the ubiquitinated proteins are extracted and degraded by the ubiquitin-proteasome system, leading to the disruption of multiple MOM [56], subsequently exposing and ubiquitinating the MIM proteins. Third, the polyubiquitin chains conjugated by Parkin recruit autophagy adaptors to initiate the engulfment of mitochondria by autophagosomes. Autophagy adaptors recognize polyubiquitin chains on the mitochondria through their ubiquitin-binding domains and interact with the microtubule-associated protein-light chain 3 (LC3) on autophagosomal membranes via their LC3-interacting region (LIR). Autophagosomes then merge with lysosomes for subsequent degradation, forming the classical PINK1/Parkin-mediated mitophagy [44, 56, 57]. Five autophagy adaptors are associated with ubiquitin-dependent mitophagy in mammalian cells: SQSTM1/p62, optineurin (OPTN), nuclear domain 10 protein 52 (NDP52), TAX1BP1, and AMBRA1 [58]. Among them, the pro-autophagic molecule AMBRA1 has been defined as a regulator of mitophagy in both canonical PINK1/Parkin-dependent and -independent systems [59–62]. Strappazzon et al. [60] demonstrated for the first time that AMBRA1 has a LIR motif used for its binding to the autophagosome adapter LC3, which is not only responsible for the enhancement of Parkin-mediated mitochondrial clearance but also drives the process of depolarized organelles in a Parkin- or p62-independent manner. However, the underlying molecular mechanisms still need to be further explored. AMBRA1 could interact with ATAD3A and promote PINK1 stability, thus controlling PINK1/Parkin-dependent mitophagy [62]. Moreover, Rita et al. [61] identified that the E3-ubiquitin ligase HUWE1 acts as a key inducing factor in AMBRA1-mediated mitophagy, a process that takes place independently of the PINK1/Parkin pathway. Parkin-assembled ubiquitin chains can also recruit autophagy adaptors in complexes with a multifunctional protein kinase complex called TANK-binding kinase 1 (TBK1), which directly phosphorylates Ras-associated protein 7A (RAB7A) and promotes mitophagy through the PINK1/Parkin pathway [63]. Additionally, TBK1 regulates the phosphorylation states of SQSTM1/p62, OPTN, and NDP52, thereby enhancing their binding affinity to the ubiquitin chains and promoting mitochondrial elimination [64]. Collectively, PINK1 functions as a molecular sensor for mitochondrial health, continuously monitoring the condition of the mitochondria until damage occurs, after which it sends a signal to recruit and activate Parkin [65].

#### Parkin-independent but ubiquitin-dependent mitophagy

**Fig. 2:**
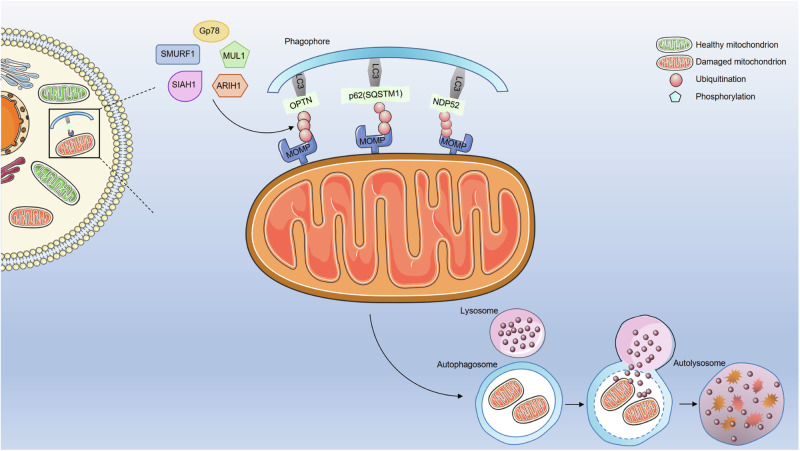
Parkin-independent but ubiquitin-dependent mitophagy pathways. The molecular mechanism of ubiquitin-dependent but Parkin-independent mitophagy is that other E3-ubiquitin ligases, such as Gp78, SMURF1, SIAH1, MUL1, and ARIH1, ubiquitinate mitochondrial proteins and trigger the recruitment of autophagy adaptors, which interact directly with autophagosomal LC3 through their LIR motifs to induce mitophagy.

Other E3-ubiquitin ligases, besides Parkin, that are involved in the regulation of mitophagy include glycoprotein 78 (Gp78) [36], smad-ubiquitin regulatory factor 1 (SMURF1) [37], seven in absentia homolog 1 (SIAH1) [38], mitochondrial E3-ubiquitin ligase 1 (MUL1) [39, 40], ariadne RBR E3-ubiquitin protein ligase 1 (ARIH1) [41, 66], among others (Fig. 2). The molecular mechanism is mainly to ubiquitinate mitochondrial proteins and trigger the recruitment of autophagy adaptors, which interact directly with LC3 through their LIR motifs to induce mitophagy. This suggests that autophagy adaptors can function independently of Parkin, but the presence of Parkin could enhance the PINK1-induced signal pathway and eventually enhance mitophagy [67]. These pathways, which occur in the absence of Parkin and independently of PINK1 kinase activity, may serve as putative drug targets for diseases involving mutations in the PINK1 kinase domain [44].

### Receptor-mediated mitophagy

**Fig. 3:**
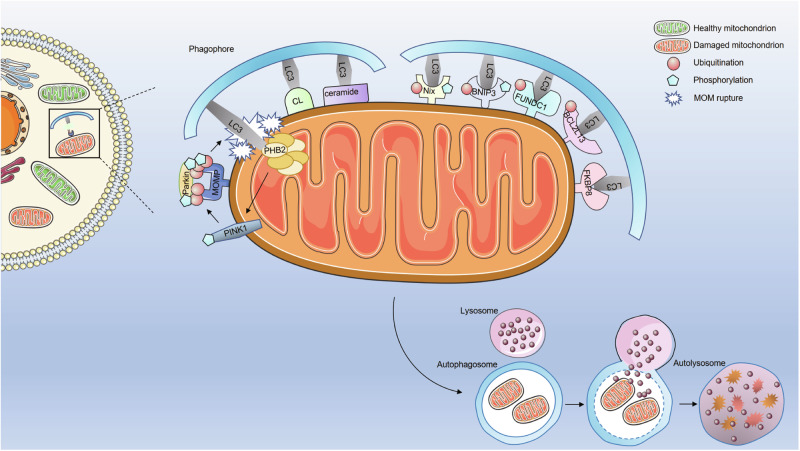
Receptor-mediated mitophagy pathways. Ubiquitin-independent mitophagy pathways primarily depend on receptor molecules that directly interact with LC3 to initiate mitophagy without undergoing ubiquitination. These receptors mainly include NIX/BNIP3L, BNIP3, FUNDC1, cardiolipin, and ceramide. The activity of mitophagy receptor proteins can be regulated through phosphorylation and dephosphorylation.

Ubiquitin-independent mitophagy pathways depend primarily on receptors that interact directly with LC3 members or their homologs (GABARAP, GABARAPL1, and GABARAPL2), which are located in the autophagosomal membrane through their LIR motifs, thus initiating mitophagy [68]. In mammalian cells, over 10 mitophagy receptors, including Nip3-like protein X (NIX)/BCL2-interacting protein 3-like (BNIP3L), BCL2-interacting protein 3 (BNIP3), and FUN14 domain-containing 1 (FUNDC1) receptors, have been identified [69, 70] (Fig. 3).

#### BNIP3L/NIX- and BNIP3-mediated mitophagy

BNIP3L/Nix and BNIP3 belong to the proapoptotic BH3-only domain proteins of the BCL2 family, located on the MOM [71–73], which can be upregulated by hypoxia-inducible factor 1 (HIF-1) and bind to LC3 through their BH3 domain to initiate mitophagy during hypoxia [67, 74]. BNIP3L/Nix, plays a crucial role in reticulocyte maturation through mitophagy [75, 76]. In addition to reticulocytes, BNIP3L/Nix can also induce mitophagy independently of Parkin in other cells [77, 78]. Additionally, BNIP3L/Nix can be degraded by proteasomes, leading to mitophagy deficiency in both ischemic neurons and brains [79].

BNIP3, similar to BNIP3L/Nix, is also a mitophagy receptor. BNIP3 deficiency can markedly reduce mitophagy and increase cell apoptosis, resulting in renal injury [80]. Furthermore, BNIP3 inhibits PINK1 proteolytic cleavage to mediate its stabilization [81]. BNIP3 and Nix can also regulate Parkin recruitment to maintain mitochondrial homeostasis, thereby highlighting the crosstalk between the PINK1/Parkin pathway and mitophagy receptors [64].

#### FUNDC1-mediated mitophagy

FUNDC1, another prominent mitophagy receptor with three transmembrane domains, is located on the MOM [82]. FUNDC1-mediated mitophagy relies on a hydrophobic interaction between LIR and LC3, which is mainly regulated by casein kinase 2 (CK2), non-receptor tyrosine kinase, ULK1, and phosphoglycerate mutase 5 (PGAM5) [82, 83]. Moreover, FUNDC1 can coordinate mitophagy and mitochondrial dynamics by interacting with the inner membrane fusion regulator optic atrophy 1 (OPA1) and the mitochondrial fission factor dynamin 1-like (DNM1L/DRP1) [84]. FUNDC1 reportedly participates in the regulation of mitophagy through various mechanisms in multiple human diseases, such as cerebral ischemia-reperfusion (IR) injury [85], myocardial IR injury [86], hypoxic pulmonary hypertension [87], metabolic syndrome [88], intestinal IR injury [89] and cancer [82, 90], which might make it as a potential therapeutic target for preventing and treating these diseases.

#### Lipid-mediated mitophagy

Certain mitochondrial lipids, such as cardiolipin (CL) and ceramide, exhibit the ability to translocate to the MOM in response to mitochondrial stress and interact directly with LC3, which can promote the recruitment of autophagosomes and thus activate mitophagy [44, 91]. Sagar et al. [92] found that mesenchymal stem cells (MSCs) derived from high-fat diet-induced obese mice failed to sequester their damaged mitochondria into LC3-dependent autophagosomes due to a decrease in mitochondrial CL content, which was proposed as a mitophagy receptor in MSCs. Oversupply of fatty acids to cardiomyocytes (CMs) is associated with increased ceramide content and elevated risk of lipotoxic cardiomyopathy. Bekhite et al. [93] found that ceramide accumulation in CMs increased mitochondrial fission regulators and mitophagic proteins LC3B and PINK1, resulting in an increase in mitophagy.

#### Other receptor-mediated mitophagy pathways

BCL2-like 13 (BCL2L13) was identified as a mammalian homolog of Atg32 to mediate the fragmentation of mitochondria and mitophagy [94]. However, the mechanism by which BCL2L13 regulates mitophagy might be different from that by which Atg32 regulates mitophagy in yeast [44]. Murakawa et al. reported that in mammalian cells, the ULK1 complex is required for BCL2L13-mediated mitophagy [95]. FK506-binding protein 8 (FKBP8) is also a mitophagy receptor that interacts with LC3A to induce Parkin-independent mitophagy [96]. Under energetic stress, the MIM protein Prohibitin 2 (PHB2) emerges as a receptor that mediates Parkin-dependent mitophagy [97]. It can bind LC3 through its LIR domain upon mitochondrial depolarization and proteasome-dependent rupture of MOM [97]. In addition, PHB2 can form a ternary protein complex with SQSTM1/p62 and LC3, which results in LC3 being loaded onto the damaged mitochondria during cholestasis-induced mitophagy [98]. According to research by Yan et al. [99], PHB2 can affect the stability of PINK1 through the PARL-PGAM5 axis, regulating PINK1/Parkin-mediated mitophagy.

Overall, a variety of physiological and pathophysiological processes are influenced by ubiquitin-independent mitophagy pathways; however, the regulation and interaction between these pathways remain to be elucidated.

## Advances and therapeutic potential of mitophagy in gynecological malignancies

**Fig. 4:**
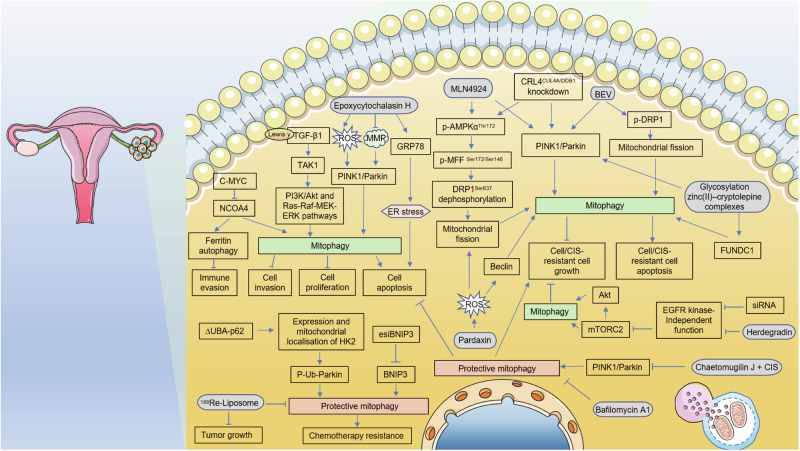
Regulation of mitophagy in OC. Under different conditions, mitophagy can exert completely paradoxical regulatory functions even in the same cell lines or animal models in OC. On the one hand, mitophagy maintains cellular mitochondrial homeostasis, promotes cell survival, and leads to resistance to chemotherapy; on the other hand, mitophagy promotes cell apoptosis and inhibits tumor cell growth.

**Fig. 5:**
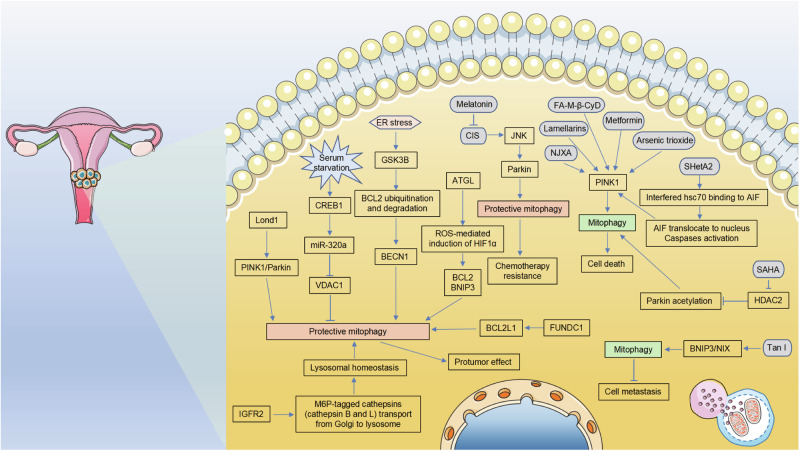
Regulation of mitophagy in CC. Similar to other types of carcinomas, mitophagy also plays a dual role in CC, i.e., protumor effect or antitumor effect. Although most of the current results were limited to in vitro conditions, these findings will undoubtedly contribute to establishing more effective integrated therapeutic regimens for CC treatment.

**Fig. 6:**
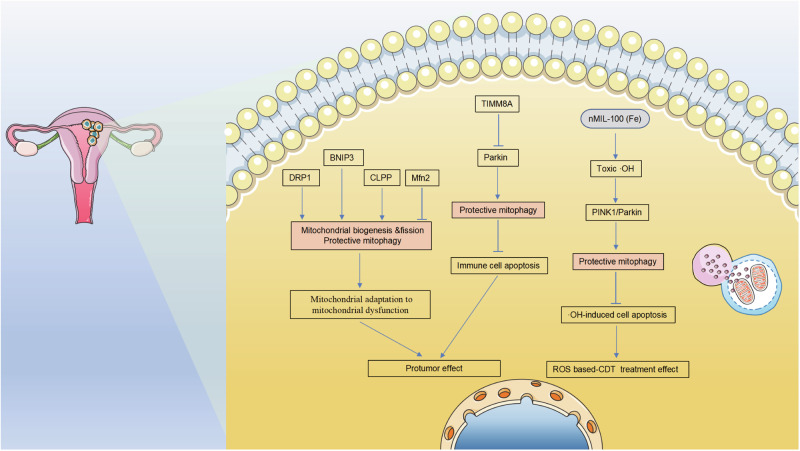
Regulation of mitophagy in EC. Research on the regulation of mitophagy in EC is limited.

The extent of mitophagy varies in different cancer types compared with that under normal conditions, highlighting the complicated connection between mitophagy and cancer [67]. Mitophagy plays a dual role in tumorigenesis; it promotes cell survival or cell death. However, the exact contribution of mitophagy to various neoplasms remains unclear. The dysregulation of mitophagy has been documented as a potential link to the pathogenesis, progression, and therapeutic resistance in gynecological cancers. In the following sections, we comprehensively summarize the roles and molecular mechanisms of mitophagy regulators in OC (Fig. 4), CC (Fig. 5), and EC (Fig. 6), and explored the possibility of targeting potential targets in mitophagy for cancer treatment.

### Mitophagy in OC

OC has the highest mortality rate and the worst prognosis among common gynecological cancers, and is currently the fifth leading cause of cancer-related deaths among women in the United States [100, 101]. Surgery combined with chemotherapy is the most common treatment strategy for OC. Despite advances in diagnostic and therapeutic techniques, treatment options for patients with OC are still limited, and the prognosis remains poor [101, 102]. Most patients at an advanced-stage relapse within a few years and develop chemotherapy resistance [103, 104], which significantly affects the treatment outcomes and prognosis. Therefore, identifying the high‐risk factors that affect cancer progression and the factors that affect chemosensitivity will be beneficial for the improvement of the prognosis of OC patients.

Abnormalities in mitophagy regulatory factors contribute to the occurrence and progression of OC and greatly impact the sensitivity of patients with OC to chemotherapy. Vianello et al. [105] demonstrated that inhibition of BNIP3-mediated mitophagy can reduce cisplatin (DDP) resistance. Meng et al. [106] reported that targeting CRL4 suppresses the growth of chemoresistant OC cells by inducing mitophagy. Depletion of the E3-ubiquitin ligase CRL4^CUL4A/DDB1^ increased mitochondrial fission by increasing AMPKα^Thr172^ and MFF^Ser172/Ser146^ phosphorylation, which in turn attracted DRP1 to mitochondria [106]. They believe that disruption of CRL4^CUL4A/DDB1^ and mitophagy may be a feasible treatment approach to overcome chemoresistance in OC, as deletion of CRL4^CUL4A/DDB1^ increased mitophagy through the PINK1/Parkin pathway and inhibited proliferation of OC cells [106]. Yu et al. [107] proved that the sensitivity of OC cells A2780 to DDP depends on mitophagy, and p62 acts as a broad autophagy receptor to regulate this process. In vivo, an experimentally induced mutation in the UBA domain of p62 could alter DDP sensitivity by enhancing the localization of HK2 on mitochondria, leading to increased phosphorylated ubiquitin levels of Parkin and stabilization of mitophagy, ultimately promoting A2780 OC cell survival and resulting in chemotherapy resistance [107].

Furthermore, Zampieri et al. [108] demonstrated that acquired resistance to DDP in human OC cells is associated with an oxidative switch that involves increased mitochondrial preservation after treatment. Therefore, targeting autophagy/mitophagy using bafilomycin A1 has been identified as a viable strategy to control the proliferation of DDP-resistant OC cells [108]. Jin et al. [109] reported that fucosylation of TGF-β1 promoted autophagy and mitophagy in OC cells by modulating the PI3K/Akt pathway and Ras-Raf-MEK-ERK pathway through TAK1. Besides apoptosis, mitophagy also has close and complex interactions with other types of cell death, such as ferroptosis and pyroptosis. Jin et al. [110] conducted a study in which they provided evidence that C-MYC exerts an inhibitory effect on ferroptosis through the mechanism of NCOA4-mediated ferritin autophagy. This inhibition subsequently leads to a reduction in ROS, suppression of mitophagy, and promotion of a malignant phenotype in OC cells.

Another factor contributing to the high mortality rate and poor prognosis in OC patients is the frequent metastases to the peritoneal cavity, characterized by the accumulation of ascites fluid and the presence of numerous tumor clusters spreading throughout the peritoneum, omentum, and serosal surfaces of various organs [111]. Peritoneal tumor-associated macrophages (TAMs) typically promote OC metastasis and suppress immune responses, which makes them a promising candidate for cancer immunotherapy [112, 113]. However, a number of tests performed on TAMs have not been successfully translated into clinical practice. Xia et al. [111] discerned two distinct subsets of TAMs in an OC peritoneal metastasis model. These subsets, characterized by differential expression of T cell immunoglobulin and mucin domain-containing 4 (Tim-4), denoted as Tim-4-positive (Tim-4+) and Tim-4-negative (Tim-4–) TAMs, exhibit phenotypic, transcriptional, ontogenic, metabolic, and functional disparities. The researchers noted that Tim-4+ TAMs demonstrate elevated levels of mitochondrial activity and mitophagy function and promote tumor growth in vivo. Moreover, mechanistic studies have shown that elevated arginase-1 levels in Tim-4+ TAMs enhance adaptive mitophagy by attenuating mTORC1 activation.

Three-dimensional spheroid formation provides a survival advantage for OC cells under conditions of peritoneal dissemination characterized by limited nutrient and oxygen availability, accompanied by a reduced metabolic phenotype and the presence of fragmented mitochondria [114]. The mouse ovarian surface epithelial (MOSE) cell model of progressive serous OC has been considered an excellent tool for identifying both cellular and molecular changes in the early and late stages of OC [115, 116]. Grieco et al. [114] used cells that differ in their capacity to induce lethal disease, as MOSE-LTICv represents a fast-developing disease that only needs 1 × 10^3^ cells to reach the endpoint, while MOSE-L cells need 1 × 10^6^ cells and ~100 days to achieve the same and therefore represent slow-developing disease. The results showed that mitochondrial fragmentation can be reversed in spheroids, and reoxygenation leads to enhanced invasion and migration in both cell types. However, only MOSE-L cells exhibited enhanced proliferation in response to reoxygenation. Known for their highly aggressive phenotype, MOSE-LTICv cells exhibit a notable degree of oxygen independence and can sustain increased levels of proliferation, migration, and invasion, even when exposed to restricted culture conditions. These aggressive cells exhibit a heightened dependence on mitophagy [114].

Drugs and small molecular inhibitors targeting mitophagy regulators and signaling pathways have been investigated in vitro and animal studies. Bevacizumab, an anti-VEGF antibody, primarily targets tumor blood vessels and exerts a cytostatic antitumor effect [117]. Kingnate et al. [118] demonstrated that bevacizumab alone increased mitophagy by upregulating PINK1 and Parkin protein expression in Caov3 OC cells. Chen et al. [119] came to a significant conclusion that the antimicrobial peptide, pardaxin effectively triggered an excessive process of mitophagy and mitochondria-mediated apoptosis in human OC cells PA-1 and SKOV3, which was achieved by stimulating ROS generation. Katreddy et al. [120] showed that the downregulation of EGFR protein, achieved through the use of siRNA or a synthetic EGFR-downregulating peptide called herdegradin, led to the induction of selective mitophagy and subsequent eradication of OC cells via stimulating the mTORC2/Akt axis, which differed from the pro-survival non-selective autophagy triggered by inhibition of EGFR kinase activity. Moreover, herdegradin induction triggered mitophagy and effectively suppressed the proliferation of orthotopic ovarian cancers in murine models [120]. Chaetomugilin J, a metabolite derived from a flowering plant named *Polygonatum sibiricum*, is a member of the Chaetomium family and demonstrates significant cytotoxic activity [121]. It can enhance apoptosis induced by DDP in human A2780 OC cells by inhibiting PINK1/Parkin-mediated mitophagy [121]. A nanomedicine, ^188^Re-liposome, can significantly inhibit the function of cancer stem cells by suppressing autophagy/mitophagy in OC cells [122]. Furthermore, it could effectively overcome drug resistance in OC, as evidenced by two case reports in which drug resistance was converted into drug sensitivity. Epoxycytochalasin H, which is also derived from *Polygonatum sibiricum*, has demonstrated the ability to hinder cell viability, disrupt mitochondrial function, initiate mitophagy, and induce the mitochondrial apoptosis pathway in A2780 OC cells [123]. Zhou et al. [124] reported that the use of glycosylation zinc(II)–cryptolepine complexes had the potential to disrupt mitophagy pathways, thereby inducing autophagy and apoptosis in SKOV3/DDP OC cells.

Although the involvement of mitophagy in OC has attracted great attention recently, current results remain limited and inconsistent. Under different conditions, mitophagy can exert completely paradoxical regulatory functions even in the same cell lines or animal models. On the one hand, mitophagy maintains cellular mitochondrial homeostasis, promotes cell survival, and leads to resistance to chemotherapy drugs; on the other hand, mitophagy promotes cell apoptosis and inhibits tumor cell growth. Extensive studies are still needed to elucidate the precise role and molecular mechanisms underlying the dysregulation of mitophagy in the initiation, progression, and resistance to chemotherapy in OC. Further, in vivo experiments and potential clinical trials are necessary to validate the antitumor effects of drugs or small molecule inhibitors targeting mitophagy.

### Mitophagy in CC

CC is one of the three most common types of malignant tumors in the female genital tract and ranks fourth in cancer-related deaths among women worldwide, with 500,000 new cases and 275,000 deaths reported annually [125, 126]. The primary risk factor linked to the development of CC is the persistent infection of human papillomavirus (HPV) [6]. The implementation of cytology screening and HPV vaccines has proven to be an effective preventive measure; however, the extent of their coverage remains inadequate across all nations, particularly in low and middle-income countries, which are characterized by the highest disease burden [127, 128]. For patients diagnosed with early-stage CC (Stage IA-IIA), surgery and/or radiotherapy are commonly advised treatment modalities as they have shown favorable clinical outcomes [129]. For patients with advanced-stage CC (Stage IIB–IV), the standard care involves radiotherapy with or without DDP-based concurrent chemotherapy; however, 5-year survival rates remain poor [129]. Therapeutics are limited because of radioresistance, chemoresistance, and postoperative recurrence; therefore, exploring the precise molecular mechanisms of the pathology and treatment resistance is of utmost exigency.

The importance of mitophagy in the onset and progression of CC has attracted wide attention in recent years. Similar to other types of carcinomas, mitophagy also plays a dual role in CC [130]. Significantly elevated expression of FUNDC1 was observed in CC tissues than in adjacent noncancerous cervical tissues [131]. The expression of FUNDC1 protein exhibited a negative correlation with the prognosis of individuals diagnosed with early-stage CC, thereby indicating its potential utility as an independent prognostic factor for both overall survival and disease-free survival. The depletion of FUNDC1 levels in CC cell lines, particularly HeLa and Caski cells, leads to a reduction in the expression of the anti-apoptotic protein BCL2L1. Additionally, it was found to inhibit the activation of LC3 when exposed to DDP and ionizing radiation. This depletion of FUNDC1 not only blocks mitophagy but also triggers the activation of caspase-3-dependent apoptosis, leading to inhibition of tumor cell proliferation and heightened cell sensitivity to DDP and ionizing radiation [131]. Li et al. [132] demonstrated that upregulation of miR-320a expression during serum starvation stress could prolong the survival of CC cells by enhancing mitophagy. In vitro experiments indicated that serum starvation-induced the expression of CREB1, which subsequently activated miR-320a expression. This activation, in turn, suppressed the expression of VDAC1, a MOM protein, in HeLa and C33A cells, leading to the promotion of mitophagy and ultimately enhancing the survival of these cancer cells. Malignant tumors often experience insufficient blood supply, resulting in an ischemic environment within the tumor tissues. Similarly, Guo et al. [133] observed a significant induction of miR-346 in HeLa cells subjected to ER stress, which was found to play a crucial role in preserving cell viability and mitigating cell apoptosis. The mechanism involved the activation of autophagy/mitophagy through the disruption of the interaction between BCL2 and BECN1, and this process was found to be dependent on GSK3B.

High-linear energy transfer (LET) heavy ions exhibit higher efficacy in triggering various biological effects, including genomic instability and cytotoxicity, in cells than conventional low-LET radiation, such as X-rays and gamma rays [134, 135]. Jin et al. [136] reported that the application of high-LET carbon ions on HeLa cells resulted in dose- and time-dependent alterations in the mitochondrial membrane potential (MMP). Cells exposed to a dose of 0.5-Gy of carbon ions displayed moderately truncated mitochondria. Subsequently, damaged mitochondria were able to be eliminated through the process of mitophagy. The inhibition of mitochondrial fission, either through the knockdown of Drp1 or FIS1 or through the use of the Drp1 inhibitor mdivi-1, suppressed mitophagy and enhanced apoptosis following irradiation at 0.5 Gy. Conversely, in cells subjected to 3-Gy radiation, mitochondria underwent division into punctate and clustered forms, which were subsequently associated with apoptotic cell death. In this particular scenario, the inhibition of mitochondrial fission resulted in mitophagy and increased cell survival. These findings reveal an innovative mechanism underlying the stress response in human CC cells, thereby offering valuable insights into the understanding of tumor development.

The insulin-like growth factor 2 receptor (IGF2R) is a type-1 transmembrane glycoprotein, characterized by a substantial N-terminal extra-cytoplasmic domain. This domain allows the receptor to interact with different ligands, represented as IGF-2 and mannose-6-phosphate (M6P)-tagged proteins. Both ligands play crucial and distinct roles in the maintenance of normal development and homeostasis in mammals [137, 138]. IGF2R has been identified as an unfavorable prognostic biomarker for patients with CC [139]. In vitro experiments revealed that IGF2R could maintain autophagy and mitophagy by regulating lysosomal homeostasis in CC cells. IGF2R depletion resulted in apoptosis, reduced cell viability, and increased susceptibility to DDP. Mechanistically, the knockdown of IGF2R disrupted the transport of M6P-tagged cathepsins (cathepsin B and L) from the Golgi to the lysosome, leading to the accumulation of abnormal lysosomes with impaired degradation capabilities. Consequently, the loss of autophagic and mitophagic functions caused the buildup of harmful ROS and aggregated proteins, ultimately resulting in apoptosis. Furthermore, the inhibition of CK2 through the administration of CX-4945 (silmitasertib) and CK2 inhibitor VIII can reduce the protein expression of matured cathepsin B and L, thereby impeding the viability of CC cells. The findings of this study indicate that potential therapeutic strategies for CC may involve targeting IGF2R itself, its M6P-tagged cargo, or the Golgi-to-lysosome transport through the use of CK2 inhibitors. Lon protease 1 (Lonp1) is an ATP-dependent protease situated within the mitochondrial matrix that plays a vital role in maintaining mitochondrial function. Wang et al. [140] demonstrated that Lonp1 expression was significantly elevated in CC tissues. Lonp1 knockdown could suppress CC cell proliferation, invasion, and migration but promote apoptosis by inhibiting PINK1/Parkin-mediated mitophagy. Recently, the importance of lipid metabolism in tumorigenesis and progression has been explored. Castelli et al. [141] confirmed the association between enhanced lipid catabolism resulting from lipase ATGL overexpression and the heightened proliferation and migration capacity of CC cells. The overexpression of ATGL in CC cell lines resulted in the induction of HIF-1α through ROS-mediated mechanisms. This induction subsequently led to an increase in the protein and mRNA levels of BCL2 and BNIP3, known targets of HIF-1α. Consequently, the activation of mitophagy occurred as a survival-promoting process.

DDP-based chemotherapy is the first-line chemotherapy used alongside radiotherapy to treat CC. However, the clinical effectiveness is limited by the emergence of drug resistance. DDP exerts its anticancer effect by inducing the mitochondrial pathway of cell apoptosis [142, 143]. Chen et al. [144] reported that DDP triggered more mitochondria to be engulfed by the lysosome, facilitated LC3-II migration on the surface of mitochondria, and upregulated mitophagy markers, such as p62, BECLIN 1, and ATG5, in HeLa cells compared with those in normal cells. Nevertheless, the administration of melatonin resulted in the reversal of these above-mentioned changes. Further studies revealed that melatonin potently inhibited the JNK/Parkin pathway activated by DDP, alleviating mitophagy and leading to more mitochondrial apoptosis in HeLa cells.

In contrast to the above studies, some evidence suggests that mitophagy exhibits antitumor effects in CC. An example is the use of folate-appended methyl-β-cyclodextrin (FA-M-β-CyD), which has been documented to exhibit targeted antitumor effects specifically in tumor cells expressing folate receptor-α (FR-α) [145]. Kameyama et al. [146] demonstrated that FA-M-β-CyD effectively entered KB cells, a subline of HeLa cells, via FR-α-mediated endocytosis. This entry resulted in a notable increase in mitochondrial transmembrane potential, a decrease in ATP production, an augmentation of ROS production, a facilitation of LC3-I conversion to LC3-II, and an induction of PINK1 protein expression. Typically, mitophagy facilitates cell survival by removing damaged mitochondria. However, mitophagy-mediated digestion of mitochondria is insufficient for survival during excessive mitochondrial ROS accumulation in cells. In aggregate, the administration of FA-M-β-CyD resulted in the induction of mitophagy-mediated cell death in KB cells, which was achieved by the excessive accumulation of mitochondrial ROS, thereby hindering tumor growth.

Lamellarins, a bioactive marine pyrrole alkaloid class, have been shown to induce mitochondrial apoptosis and reduce viability in HeLa cancer cells. An in-depth analysis of the mitochondria in cells treated with lamellarins demonstrated a change in the abundance of the two Opa1 isoforms, with a shift from Opa1-L to Opa1-S. This alteration in isoform abundance resulted in disrupted mitochondrial morphology, dissipated membrane potential, and subsequent accumulation of PINK1 and LC3-II [147]. In a comparable manner, the induction of mitophagy in HeLa cells through the PINK1/Parkin signaling pathway by metformin and/or arsenic trioxide results in mitophagic apoptosis and the suppression of tumor cell proliferation [148]. Tanshinone I (Tan I) is a lipid-soluble constituent found in *Salvia miltiorrhiza* (Danshen) that effectively impedes the metastasis of CC cells through the stimulation of BNIP3/NIX-mediated mitophagy and the reconfiguration of mitochondrial metabolism [149]. Another traditional Chinese medicine, nujiangexanthone A (NJXA), a bioactive compound derived from the leaves of *Garcinia nujiangensis*, has also been documented to impede the proliferation of CC cells and induce cellular demise through the facilitation of PINK1/Parkin-dependent mitophagy [150].

In addition to phosphorylation and ubiquitination, accumulating evidence indicates that the acetylation of the critical mitophagy machinery influences the extent of mitophagy; however, the precise mechanism remains elusive [151, 152]. The results of mass spectrometry analysis revealed that Parkin exhibits interactions with two upstream molecules, namely acetylase acetyl-CoA acetyltransferase 1 (ACAT1) and deacetylase HDAC2. Bioinformatics analysis demonstrated that HDAC1/2 was significantly upregulated in human CC tissue, whereas Parkin expression was inversely correlated with HDAC2 expression, indicating the low acetylation level of Parkin in CC. In vitro experiments demonstrated that inhibition of HDAC by administration of suberoylanilide hydroxamic acid (SAHA) resulted in the acetylation of Parkin at lysine residues 129, 220, and 349. This acetylation event subsequently triggered mitophagy, ultimately leading to the suppression of HeLa and SiHA cell proliferation [153].

Heat shock proteins (HSPs) present a logical focus for the development of novel cancer therapeutics [154]. Hsc70 has been identified as a protein that binds to SHetA2 and has the ability to sequester AIF in the cytoplasm, thereby preventing its localization in the nucleus. Analysis conducted by The Cancer Genome Atlas indicated that increased expression of hsc70 was correlated with a reduced survival rate among patients diagnosed with CC. Consistent with findings in other forms of cancer [155–157], Rai et al. [158] demonstrated that SHetA2 hindered the binding of hsc70 to AIF, resulting in the translocation of AIF to the nucleus and the activation of caspases in CC cells. However, the mechanism underlying SHetA2-induced cell death in CC cells was primarily attributed to an overabundance of PINK1/Parkin-mediated mitophagy rather than caspase-induced apoptosis. This was further confirmed through in vivo experiments, where excessive mitophagy and a lack of caspase activation were observed in SHetA2-inhibited xenograft tumors.

Although most of the current results were limited to in vitro conditions, these findings will undoubtedly contribute to establishing more effective integrated therapeutic regimens for CC treatment. However, owing to their multifaceted action mechanisms in tackling resistant cancer cells, further investigation is required to clarify the resulting non-specificity, potential toxicity in normal tissues, and detailed pharmacokinetics.

### Mitophagy in EC

EC is the most common gynecological cancer in high-income countries, with 417,000 new diagnoses made globally in 2020 [159]. The risk in women increases by approximately 0.5% annually with age, potentially owing to declining fertility and increasing body weight [160]. EC is primarily classified into two clinicopathologic subtypes: estrogen-dependent type I and estrogen-independent type II [161]. Current treatment options include surgery, radiotherapy, and chemotherapy for EC. In the context of young patients requiring fertility preservation, the use of hormonal therapy involving progestin agents has exhibited encouraging clinical efficacy in cases of early-stage EC, particularly when the estrogen or progesterone receptor shows positive expression [162]. Despite advances in modified chemotherapy, radiotherapy, and immunotherapy for gynecological malignant tumors, progress in the treatment of EC over the past decades has been limited, especially for patients afflicted with advanced-stage EC (stage III or IV) [163, 164]. Hence, it is imperative to comprehend the molecular mechanisms underlying the progression of EC and to ascertain novel therapeutic targets to enhance treatment outcomes.

The emerging role of mitochondrial alterations in EC tumorigenesis, progression, and prognosis is of current interest [165]. Most type I EC tumors have been reported to exhibit pathogenic somatic mutations in mitochondrial DNA, which are linked to alterations in respiratory complex I, mitochondrial biogenesis (oncocytic-like phenotype), and an augmented antioxidant response [166, 167]. Cormio et al. [168] first reported an upregulation of fission protein Drp1, mitophagy protein BNIP3, mitochondrial protease CLPP, antioxidant and anti-apoptotic protein ALR, and Bcl-2, alongside a downregulation of fusion protein Mfn2 in type I EC tissues exhibiting impaired complex I when compared to control and hyperplastic tissues. These findings suggest that individuals with type I EC, specifically those with mutations in mitochondrial DNA and deficiencies in complex I, may experience activation of mitochondrial biogenesis, as well as fission, mitophagy, and proteolysis processes. These activations are likely aimed at maintaining a sufficient quantity of functional mitochondria to withstand mitochondrial dysfunction and ensure survival.

Mounting evidence indicates that immune dysfunction is implicated in EC pathogenesis. Zhu et al. [169] conducted a pan-cancer analysis of TIMM8A and found that its overexpression was significantly associated with poor prognosis and immune infiltration, including CD8 + T cells, Th2 CD4 + T cells, and M2 macrophages in EC. Further analysis revealed a negative correlation between highly expressed TIMM8A and Parkin, resulting in the inhibition of mitophagy and induction of immune cell apoptosis, contributing to tumorigenesis. However, those findings are merely based on comprehensive bioinformatic analysis. Further research to evaluate the changes in the immune function of those immune cells under TIMM8A knockdown or overexpression is needed, which may provide inspiration for future drug development for EC.

In recent times, several nanoplatforms have been employed in cancer therapy, specifically chemodynamic therapy (CDT), by integrating endogenous and exogenous stimuli-responsive ROS production. CDT exploits the inherent biochemical characteristics of the unique TME to convert intra-tumoral H_2_O_2_ into more potent oxidative ·OH species. This conversion is achieved by initiating and facilitating diverse catalytic reactions, such as Fenton or Fenton-like chemistry, within the TME itself [170]. The nanoscale metal-organic frameworks (nMOFs), specifically nMIL-100 (Fe), which possess abundant Fe active sites, were chosen as Fenton nanocatalysts to increase intracellular reactive ·OH stress in EC cells [171]. The findings of this study suggest that the combination of H_2_O_2_ with nMIL-100 (Fe) nanoparticles resulted in increased cytotoxicity towards EC cells, particularly the progesterone treatment-insensitive KLE cells. It was observed that the highly toxic ·OH generated by nMIL-100 (Fe) nanoparticles could activate the classical PINK1/Parkin pathway-mediated cytoprotective mitophagy, thereby partially mitigating ·OH-induced apoptosis. In addition, the application of siRNA-mediated Parkin knockdown and the inclusion of the mitophagy inhibitor Mdivi-1 as pretreatments for EC cells proved effective in facilitating the synergistic effect of nMIL-100 (Fe) in conjunction with H_2_O_2_-induced oxidative damage. These results suggest that the extent of mitophagy should be taken into account to enhance the effectiveness of ROS-based CDT for EC treatments.

Research on the regulation of mitophagy in EC is limited. Although the current understanding of the clinical utility of mitochondrial dynamics, biogenesis, and mitophagy as biomarkers for the onset and progression of EC is still in its nascent stages, it represents great potential. Consequently, additional research endeavors are necessary to further explore this area.

## Conclusion and future perspectives

Gynecological malignancies impact the health and quality of life of affected women. Despite the emergence of novel targeted therapeutic drugs in recent years, the overall survival for these patients has not improved significantly, and instances of recurrence and drug resistance remain prevalent, particularly in patients with OC. Therefore, the mechanisms underlying the onset and progression of gynecological malignancies need to be comprehensively investigated to provide substantial evidence for developing more effective treatment modalities to improve patient prognosis.

Accumulating evidence highlights the involvement of aberrant mitophagy in gynecological malignancies, which has diverse effects on tumorigenesis, tumor progression, and treatment sensitivity. Furthermore, studies have demonstrated the promising effects of certain drugs or inhibitors on inhibiting tumors or enhancing treatment sensitivity. These findings signify the progress in mitophagy research in gynecological malignancies but also underline the challenges ahead. The study of mitophagy in gynecological malignancies remains an ongoing and complex endeavor. Mitophagy might be a new entry point for targeted intervention in gynecological malignancies in the future, but the dual roles and intricate mechanisms of mitophagy in tumorigenesis and drug resistance in gynecological malignancies require further exploration and clarification. It is believed that as research in this field deepens, more specific mechanisms of mitophagy can be discovered, and effective targeted drugs can be developed to provide new strategies for the treatment of gynecological malignancies.
